# Tenofovir Hot-Melt Granulation using Gelucire^®^ to Develop Sustained-Release Vaginal Systems for Weekly Protection against Sexual Transmission of HIV

**DOI:** 10.3390/pharmaceutics11030137

**Published:** 2019-03-20

**Authors:** Fernando Notario-Pérez, Raúl Cazorla-Luna, Araceli Martín-Illana, Roberto Ruiz-Caro, Juan Peña, María-Dolores Veiga

**Affiliations:** 1 Departamento de Farmacia Galénica y Tecnología Alimentaria, Facultad de Farmacia, Universidad Complutense de Madrid, 28040 Madrid, Spain; fnotar01@ucm.es (F.N.-P.); racazorl@ucm.es (R.C.-L.); aracelimartin@ucm.es (A.M.-I.); rruizcar@ucm.es (R.R.-C.); 2 Departamento de Química en Ciencias Farmacéuticas, Facultad de Farmacia, Universidad Complutense de Madrid, 28040 Madrid, Spain; juanpena@ima.ucm.es

**Keywords:** controlled release, ex vivo bioadhesion, Gelucire^®^, Human Immunodeficiency Virus, mucoadhesive vaginal compacts, Tenofovir

## Abstract

Hot-melt granulation is a technique used to obtain granules by dispersing a drug in polymers at a high temperature. Tenofovir, an antiretroviral drug with proven activity as a vaginal microbicide, was dispersed in melted Gelucire^®^ (or a mixture of different Gelucire^®^) to obtain drug-loaded granules. Studies performed on the granules proved that the drug is not altered in the hot-melt granulation process. The granules obtained were included in a matrix formed by the hydrophilic polymers hydroxypropylmethylcellulose and chitosan to obtain vaginal tablets that combine different mechanisms of controlled release: The Gelucire^®^ needs to soften to allow the release of the Tenofovir, and the hydrophilic polymers must form a gel so the drug can diffuse through it. The studies performed with the tablets were swelling behavior, Tenofovir release, and ex vivo mucoadhesion. The tablets containing granules obtained with Tenofovir and Gelucire^®^ 43/01 in a ratio of 1:2 in a matrix formed by hydroxypropylmethylcellulose and chitosan in a ratio of 1.9:1 were selected as the optimal formulation, since they release Tenofovir in a sustained manner over 216h and remain attached to the vaginal mucosa throughout. A weekly administration of these tablets would therefore offer women protection against the sexual transmission of HIV.

## 1. Introduction

The hot-melt granulation technique consists of dissolving or dispersing an active principle in one or more melted polymers in order, after cooling, to obtain granules with a defined structure [1]. The melt entropy of the different components determines whether they will crystallize simultaneously, in turn conditioning the microstructure of the granules in the solid state [2].

The most common applications of this technique in pharmaceutical development are targeted drug release, masking the taste of active substances, and particularly improving the solubility of poorly soluble drugs [3]. In the latter case, solid dispersions of the drug are usually prepared in an inert hydrophilic matrix, causing the active principle to convert from its crystalline state to an amorphous form and improving its solubility [4]. However, this technique could also be applied in reverse to delay the dissolution of water-soluble drugs, which would aid the development of sustained-release systems capable of releasing the drug at a given rate, thereby achieving a constant concentration of the active principle at the site of action [3,4]. Some references to this possibility can be found in the literature: Mini-matrices have been developed where the drug is found in polymers as diverse as ethyl cellulose and xanthan gum [5], polyvinylpyrrolidone (PVP) [6], thermoplastic polyurethane [7] or ethylene vinyl acetate and polyethylene oxide [8]. One alternative yet to be explored would be to develop these systems using as carriers various Gelucire^®^, a group of amphiphilic excipients commonly used to develop sustained-release matrices [9,10]. Another advantage is that Gelucire^®^ is commercialized with different melting points and hydrophilic-lipophilic balances (HLB), offering a wide range of options from which to select the most suitable for our purposes [11].

This last option—developing systems for the sustained release of drugs—raises the possibility of applying this technique to produce a vaginal microbicide to prevent the sexual transmission of the human immunodeficiency virus (HIV). A vaginal microbicide is any suitably formulated compound which, when applied in the vagina before intercourse, has the ability to prevent or reduce the transmission of sexually-transmitted diseases. The latest published data reflect the gender inequality in HIV protection, with an estimated 18.6 million women and girls living with the virus, and three young women infected every four minutes [12]. This explains the growing interest in recent decades in developing this type of formulations as a tool to protect women from the sexual acquisition of HIV.

Tenofovir (TFV) is an antiretroviral drug, specifically a nucleotide analogue reverse transcriptase inhibitor, approved by the Food and Drug Administration and recommended by the World Health Organization for its use as a first-line drug for the treatment of HIV [13]. It has also been extensively studied for the development of vaginal microbicides, mainly since the publication in 2010 of the results of the CAPRISA 004 trial, in which a 1% TFV gel showed a 39% effectiveness in protecting against HIV infection [14]. However, the fact that it required a daily application reduced the effectiveness of the formulation, so the current trend is to develop sustained-release formulations that require less frequent administration [15]. TFV is a hydrophilic drug that is soluble in aqueous media, such as vaginal fluid, requiring techniques to obtain sustained-release formulations that slow its dissolution rate in the vaginal environment.

The aim of this work is to prepare granules of TFV by hot-melt granulation using one or more Gelucire^®^ containing mono-, di- and triglyceride esters of fatty acids (C8 to C18), –Gelucire^®^ 39/01 (G39) and Gelucire^®^ 43/01 (G43)–, which are characterized by their low HLB value (HLB = 1) and low melting point (39 °C and 43 °C respectively). However, rather than manufacturing matrices, the granules are incorporated in a hydrophilic matrix formed by a combination of hydroxypropylmethyl cellulose (HPMC) and chitosan (CH), two polymers that are capable of swelling in the presence of vaginal fluid and have proven to be an excellent choice for the development of sustained drug-delivery systems [16]. Vaginally administered tablets could then be developed in which the release of the drug would be conditioned by multiple factors. A Gelucire^®^ with a predominantly lipophilic rather than hydrophilic character would prevent the dissolution of the granules in the vaginal medium, while their low melting point would cause Gelucire^®^ to soften at body temperature and allow the vaginal fluid to diffuse through it and dissolve the drug; alternatively, the combination of HPMC and CH would swell with the vaginal fluid and form a gel which would initially delay the arrival of the vaginal fluid at the granules, and subsequently hinder the diffusion of the dissolved drug through the gel to reach the vaginal environment. The aim is to develop mucoadhesive systems that offer weekly protection against HIV infection and encourage greater adherence to the use of the microbicide, due to their lower frequency of administration, and consequently achieve greater protective efficacy.

## 2. Materials and Methods

### 2.1. Materials

Gelucire^®^ 39/01 (G39, lot: 3E3701-2) and Gelurice^®^ 43/01 (G43, lot: 1E5203-2) were a gift from Gattefossé (Saint-Priest, France). Tenofovir (TFV, lot: FT104801401) was supplied by Carbosynth Limited (Berkshire, UK). Chitosan, whose properties were characterized experimentally determining that it has a molecular weight of 32.1 kDa—being therefore a low-molecular-weight chitosan-, with an intrinsic viscosity of 24.75 dL/g and a degree of deacetylation of 54. 7% [17] (CH, lot: 0055790), was provided by Guinama (La Pobla de Vallbona, Spain). Hydroxypropylmethylcellulose—Methocel^®^ K 100 M (HPMC; lot: SB13012N31) was kindly supplied by Colorcon Ltd. (Kent, UK). Anhydrous calcium hydrogen phosphate—Emprove^®^—(ACDP; lot: K93487944416) was supplied by Merck (Darmstadt, Germany). Polyvinylpyrrolidone—Kollidon^®^ 30—(PVP; lot: 98-0820) was purchased from BASF Aktiengesellschaft (Ludwigshafen, Germany). Magnesium stearate PRS-CODEX (MgSt; lot: 85269 ALP) was acquired from Panreac (Barcelona, Spain).

All other reagents in this study were of analytical grade and used without further purification. Demineralized water was used in all cases.

### 2.2. Preparation of the Granules

The granules were prepared using G39, G43 and a mixture of both in equal proportions in the carrier/TFV ratios, shown in Table 1. The granules were obtained by first melting the Gelucire^®^ in an oil bath (Selecta^®^ Univeba-401, Barcelona, Spain), taking care to ensure the temperature never exceeded 50°C. Once completely melted, the TFV was incorporated and the mixture was stirred until the drug was homogeneously dispersed. The mixture was then spread over a smooth non-stick surface and left to stand at room temperature for a few minutes to solidify. Finally, the solidified mixture was forced through a 1 mm mesh to obtain the granules. Thus, this ensured that all granules obtained had a size of < 1mm in diameter.

### 2.3. Characterization of the Granules

#### 2.3.1. Infrared Spectroscopy

Fourier Transform Infrared Attenuated Total Reflection Spectroscopy (FTIR-ATR) was used to characterize the pure materials and prepared granules with a Perkin-Elmer spectrophotometer equipped with a MIRacle™ accessory designed for FTIR-ATR measurements (Perkin-Elmer, Waltham, MA, USA).

#### 2.3.2. Thermal Analysis

Hot-stage microscopy studies were performed between 30 °C and 350 °C. Approximately 1 mg of each sample was placed on a microscope slide with a cover on a Kofler stage and heated at a rate of 2 °C/min. A thermogalen microscope fitted with the Kofler stage (Leica, Wetzlar, Germany) was used for the microscopy examinations.

Thermogravimetric analysis (TGA) and differential scanning calorimetry (DSC) were done in an SDT-Q 600 TA Instruments TG/DTA analyzer (TA Instruments, New Castle, DE, USA). In this case, about 5−10 mg of samples were placed in a pinholed aluminium sample pan with a lid and heated in atmospheric air to between 30 °C and 500 °C at a rate of 10 °C/min.

#### 2.3.3. X-ray Diffraction

An automated Philips X’Pert-MPD X-ray diffractometer with Bragg–Brentano geometry (Malvern Panalytical Ltd., Royston, UK) was used to obtain the X-ray diffraction patterns of pure materials and all granulated systems. Monochromatized Cu-Kα radiation (λ = 1.5406 Å) was irradiated over the samples at 45 kV, 40 mA and a time per step of 2 s, and analyzed between 2θ angles of 5° and 50°. 

An X-ray thermodiffraction of the TFV was also performed using an X’Pert PRO MPD in theta-theta configuration with an Anton Paar HTK1200 high-temperature camera (Malvern Panalytical Ltd., Royston, UK). Monochromatized Cu-Kα radiation (λ = 1.5406 Å) was irradiated over the sample at 45 kV and 40 mA. The sample was heated to 5 °C/min and analyzed between 2θ angles of 5° and 50° at 25 °C, 150 °C, 175 °C and 225 °C. The sample was then cooled at the same rate and analyzed at 200 °C, 150 °C and 25 °C under the same conditions.

### 2.4. Preparation of the Tablets

Vaginal tablets were developed with a combination of hydrophilic polymers (HPMC and CH) containing the granules developed previously. Each granulated system was in turn included in three different combinations of HPMC and CH. HPMC, CH and ACDP were mixed, and the mixture was then wetted with a solution of PVP in ethanol to produce a mass, which was granulated using a 1 mm mesh. Three granulates were obtained (C1, with a ratio of 1:1 HPMC/CH; C2, with a ratio of 1.9:1 HPMC/CH; and C3, with a ratio of 1:1.9 HPMC/CH) and dried at room temperature for 24 h. Lastly, each granulate was mixed with the TFV granules for a final content of 30 mg of drug and 290 mg of hydrophilic polymer per tablet. All granulates were prepared by forcing them through the same mesh to ensure that all granules—polymeric and TFV-loaded granules—had the same size (<1 mm in diameter), and the blends were homogeneous. MgSt was added to the total dried granules before tableting. The final content of each batch is shown in Table 2.

The tablets were produced using a press similar to the one used for preparing solid samples for analysis by IR spectroscopy. A constant pressure of 5 tons was applied for four minutes using a punch. The resulting tablets were cylindrical in shape with a diameter of 13 mm and a height of 2.5–2.8 mm.

### 2.5. Assessment of the Tablets

#### 2.5.1. Swelling Behavior

The method described by Ruiz-Caro et al. [18] was used to analyze the swelling behavior of each batch in simulated vaginal fluid (SVF) [19]. The test was done in triplicate in a shaking water bath (Selecta^®^ UNITRONIC320 OR, Barcelona, Spain) at 37 °C and 15 opm. The discs were removed from the medium at predetermined times, placed on filter paper to eliminate excess liquid and weighed. The swelling ratio (SR) was calculated following Equation (1) where *T_s_* and *T_d_* correspond to the swollen and dry tablet weights respectively:

(1) $$SR=\frac{T_{s}-T_{d}}{T_{d}}\cdot 100.$$

Since the initial tablet weight varies depending on the amount of Gelucire^®^ it contains, the *SR* of different batches was compared by determining the adjusted swelling ratio (*ASR*), where the weight increment is related to the amount of swellable polymer, as shown in Equation (2) where *SR*, *T_d_* and *SP* correspond respectively to the swelling ratio determined by Equation (1), the dry tablet weight, and the amount of swellable polymer in the tablet:

(2) $$ASR=\frac{SR\cdot \;T_{d}}{SP}.$$

#### 2.5.2. Release Study

The release of TFV from the batches was evaluated with the method described by Sánchez-Sánchez et al. [20]. The tablets were immersed in 80 mL of SVF in a borosilicate glass bottle and placed in a shaking water bath (Selecta^®^ UNITRONIC320 OR, Barcelona, Spain) at 37 °C and 15 opm. The test was performed in triplicate. Release tests are performed in sink conditions because the solubility of TFV in SVF, measured at room temperature, is 4 mg/mL. 5 mL samples were taken and filtered at given times, and the medium was replaced with the same volume of SVF at the same temperature. The TFV released was quantified by UV spectroscopy at a wavelength of 260 nm in a Shimadzu^®^ UV-1700 (Kyoto, Japan). 

The similarity factor (*f*_2_) (Equation (3)) was calculated in order to statistically demonstrate the differences between the batches.

(3) $$f_{2}=50\times \log\{{\left[1+\left(\frac{1}{n}\right)\sum_{j=1}^{n}W_{j}{\left|R_{j}-T_{j}\right|}^{2}\right]}^{-0.5}\times 100\}.$$

This is a model-independent index described by Moore and Flanner [21], where *n* is the number of samples for each dissolution test, *R_j_* and *T_j_* are the drug release percentage at each time for the reference and test product respectively, and *W_j_* is a weight factor, which in this study is equal to 1. A value of *f*_2_ > 65 means similarity between the profiles of over 95%, while *f*_2_ < 65 indicates that the profiles are significantly different [22].

Finally, the experimental drug-release data were fitted to different model-dependent methods (zero order, first order, Higuchi, Hixson-Crowell, Hopfenberg and Korsmeyer-Peppas models) to investigate the drug release kinetics [23].

#### 2.5.3. Mucoadhesion Test

The method described by Notario-Pérez et al. [24] was used to evaluate ex vivo mucoadhesion time. A sample of freshly excised veal vaginal mucosa was obtained from a local slaughterhouse and attached to an 8.5 cm × 5 cm stainless steel plate with a cyanoacrylate adhesive. Each tablet was then placed over the mucosa and weight of 500 g was applied for 30 s. The system was placed at an angle of 60° in a beaker containing 150 mL of SVF and then in the shaking water bath (Selecta^®^ UNITRONIC320 OR, Barcelona, Spain) at 37 °C and 15 opm. All batches were tested in duplicate.

## 3. Results and Discussion

### 3.1. Characterization of the Granules

#### 3.1.1. Infrared Spectroscopy

The analysis of the granules by FTIR-ATR, and the subsequent comparison with the spectrum obtained by analyzing the raw materials used in their preparation, reveal any possible interactions that may have occurred at the molecular level during the granulation process. These interactions—if they occur—require an in-depth analysis to ensure that the therapeutic efficacy of the TFV has not been modified.

**Figure 1:**
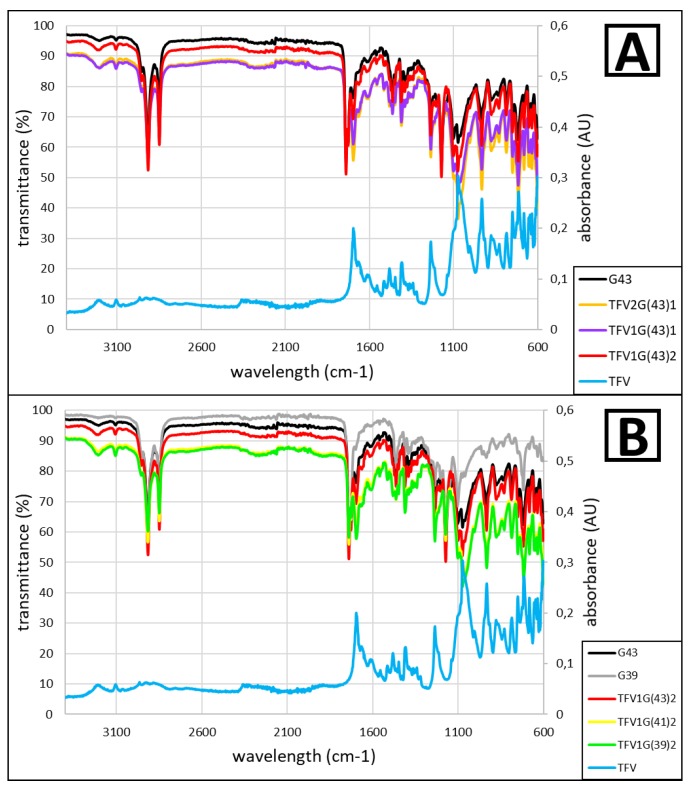
FTIR-ATR spectra of TFV, G43 and their combinations (**A**); and TFV, G43, G39 and their combinations in a 1:2 ratio of TFV/Gelucire^®^ (**B**). The absorbance spectra (right axes) correspond to TFV.

The two types of Gelucire^®^ analyzed (G39 and G43) showed a very similar FTIR-ATR spectrum, which is to be expected given their close structural similarity (Figure 1B). These spectra are also clearly similar to those obtained by other authors with other types of Gelucire^®^ [25]. Characteristics worth noting include a band at around 1650 cm^−1^ corresponding to the C–O bonds and another double band around 2800 cm^−1^, due to the C=O bond [26].

TFV is notable for a band around 1700 cm^−1^, corresponding to C=O stretching [27]. It also has bands at 3100 cm^−1^ and 3200 cm^−1^ corresponding to C–H and N–H bonds respectively [28] (Figure 1).

The results of the spectra obtained with the prepared granules are in all cases very similar, and always resemble the results that would be obtained by superimposing the spectra for the raw materials (Figure 1). 

The FTIR-ATR analysis confirms that the raw materials—Gelucire^®^ and TFV—do not interact with each other at a molecular level during the granulation process, thus maintaining the drug’s properties.

#### 3.1.2. Thermal Analysis

**Figure 2:**
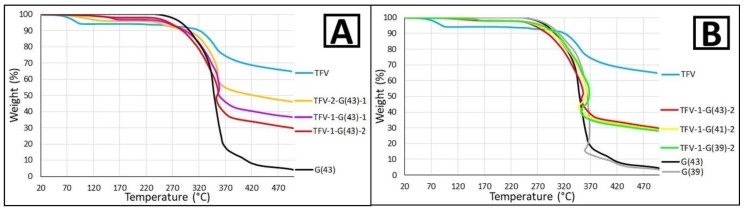
Thermogravimetric analysis (TGA) curves of TFV, G43 and their combinations (**A**); and TFV, G43, G39 and their combinations in a ratio of 1:2 of TFV/Gelucire^®^ (**B**).

Thermal analysis was performed in order to determine the characteristics of the granules in greater detail. The TGA analysis (Figure 2) shows the weight lost by the granules during heating. The raw materials were also analyzed using this technique. In the case of TFV, about 5% of the weight is lost at around 90 °C (this is probably a loss of the water captured by the drug during storage), followed by a strong degradation that possibly corresponds to the drug decomposition, beginning at around 320 °C. At 500 °C, the sample maintains about 65% of its initial weight. In contrast, the Gelucire^®^ samples show no water loss in the TGA analysis, due to the Gelucire^®^’s limited uptake of water at ambient humidity conditions [29], and there is no change in the TGA curves until around 240 °C. From this point until 370 °C the samples undergo a marked decomposition and lose over 80% of their weight. With further heating, Gelucire^®^ samples continue losing weight more slowly and when the test ends at 500 °C, less than 5% of the initial weight remains. Both G43 and G39 show similar results, but should be noted that the final weight loss is lower in the G39 sample. These minor differences between the different Gelucire^®^ products can be attributed to the slight variations in their composition [11]. Granules combining TFV and Gelucire^®^ in all cases display an intermediate behavior between the raw materials used in their manufacture. The comparison of granules with different TFV/Gelucire^®^ ratios show that the greater the amount of Gelucire^®^ included in the sample, the higher the final weight loss (Figure 2A). The variations are almost negligible in granules prepared with different Gelucire^®^, although the inclusion of more G39 than G41 can still be seen to cause the granules to degrade more, as in the case of the raw materials. Finally, it should also be noted that no weight loss is observed in the TGA analysis of the granules at around 90 °C, as occurs in the TFV sample, which confirms that the weight loss is due to water, as the raw materials are heated and the water has already been removed during the preparation of the granules.

**Figure 3:**
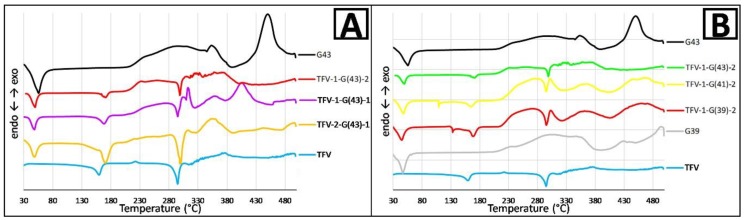
Differential scanning calorimetry (DSC) curves of TFV, G43 and their combinations (**A**); and TFV, G43, G39 and their combinations in a ratio of 1:2 of TFV/Gelucire^®^ (**B**).

DSC analyses were performed to check if during the process of preparation of the solid dispersion it occurs the dissolution of the active principle in the molten vehicle, which would later be reversible by crystallization of the TFV during the cooling of the binary system, and the results are shown in Figure 3. The TFV DSC curve is characteristic, due to an endothermic peak at around 160 °C and an exothermic peak at around 220 °C, which may correspond to changes in the crystallinity of the drug. A stronger endothermic peak beginning at 295 °C corresponds to the TFV melting point [30]. Finally, drug decomposition is observed at around 315 °C. 

An endothermic peak can be seen in both G43 and G39 at approximately their respective melting points. These melting points begin shortly after ambient temperature, as has already been observed by other researchers [31], and can be explained by the fact that Gelucire^®^ are multicomponent mixtures with a semi-solid nature. Exothermic readings beginning at 230 °C are observed in both Gelucire^®^, and the main difference is in their decomposition, which in both cases begins after 400 °C but is much more vigorous for G43 than for G39.

When analyzing the DSC curves from the granules combining one or both Gelucire^®^ with TFV, the main observation is an intermediate curve between the curves obtained from the raw materials. The three characteristic peaks of TFV appear in all the prepared granules, so mixing with Gelucire^®^ apparently has no effect on the drug. Nevertheless, it should be noted that the inclusion of TFV appears to lower the melting point of Gelucire^®^ (Figure 3). 

Finally, hot stage microscopy (HSM) was performed to complete the thermal analysis of the samples. TFV can be seen as a crystalline substance in the visualization. When heating the sample, some changes in the shape of particles are observed at 167 °C. The temperature continues rising and a similar behavior can be seen on reaching 220°C. These two changes may correspond to crystalline transitions of TFV, as was assumed to occur when analyzing the DSC curve. Finally, the sample begins to melt at around 265 °C.

Both Gelucire^®^ appear as irregularly-shaped particles of a waxy substance with a rough surface. They are crystalline, since polarized light can pass through the samples. The start of the melting process is identified by HSM at a somewhat lower temperature than indicated by the supplier (40 °C for G43 and 36 °C for G39), as shown in the DSC analysis (Figure 3). G39 turns brown at around 310°C, as though the sample had charred, and it appears to start boiling.

**Figure 4:**
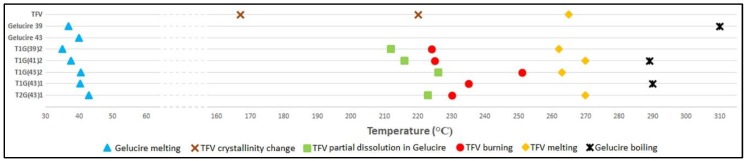
Diagram of the changes visualized during the observation of TFV, Gelucire^®^ and the granules by hot stage microscopy.

Very similar results are obtained when analyzing the granules by HSM. The main changes are summarized in Figure 4. They all appear in the microscope as irregular particles that allow the partial passage of polarized light. In all the samples, the first change observed is the melting of the Gelucire^®^. The more G39 there is in the granules, the lower the melting point (Figure 4). When comparing samples containing only G41 and TFV, the granules with twice as much TFV as G41 have a higher melting point. However, the melting of G41 is more difficult to visual identify, due to its low proportion, so it probably occurs before it can be visually detected (when a halo of melted Gelucire^®^ is seen around the TFV particles). Once the Gelucire^®^ is completely melted, there are more particles that allow the passage of polarized light, corresponding to TFV crystals surrounded by melted Gelucire^®^. The first crystalline transition in the TFV raw material is not detected in any of the granules. However, an interesting phenomenon occurs at the temperature of the second crystalline transition of TFV. From 212 °C to 226 °C, depending on the sample, the TFV crystals that allow the passage of polarized light to disappear, probably because after the change in its crystallinity TFV is able to dissolve into the melted Gelucire^®^. This ability of melted Gelucire^®^ to dissolve drugs has also been noted by other researchers [31]. However, some TFV crystals are still in evidence, so the amount of molten Gelucire^®^ is insufficient to dissolve all the TFV present in the sample. A little later, at between 224 °C and 251°C, the sample carbonizes and turns brown, probably because the dissolved TFV has burned. Between 262–270 °C, the remaining particles that still allow the passage of polarized light disappear completely in some samples, corresponding to the melting of the undissolved TFV, which occurs at the temperature at which the TFV raw material melts. Finally, at around 290 °C, the Gelucire^®^ can be seen to be boiling. After analyzing all the information from the thermal analysis of the granules, it can be confirmed that no interaction between Gelucire^®^ and TFV occurs at the manufacturing temperatures; however, a more in-depth study was made using X-ray diffraction techniques to confirm the TFV transitions and some of the interactions that are assumed to occur at higher temperatures.

#### 3.1.3. X-ray Diffraction

X-ray spectrometry studies were performed to evaluate if these is any change in the crystalline state of the drug during the granulation process. These studies could also corroborate the hypotheses formulated after the thermal analysis of the granules. First the raw materials (TFV, G43 and G39) and the manufactured granules were analyzed to compare them and confirm that there is no interaction between the drug and the carriers during granulation.

**Figure 5:**
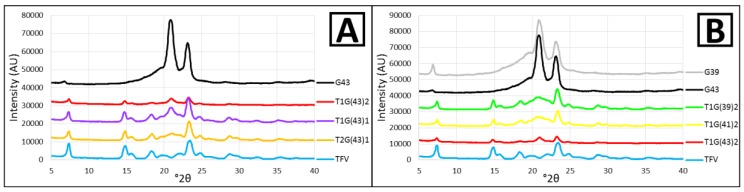
X-ray diffraction patterns of pure TFV, G43 and their combinations (**A**); and TFV, G43, G39 and their combinations in a ratio of 1:2 TFV/Gelucire^®^ (**B**).

The X-ray diffraction pattern for TFV reveals the four characteristic peaks of the drug at 7.3, 14.7, 18.3 and 23.4° 2θ [32]. This clearly confirms that TFV is a crystalline substance, as observed in HSM. There are hardly any differences between G43 and G39, and both are crystalline (as observed in HSM) with two intense peaks at 20.9 and 23.1° 2θ. This agrees with the results obtained for other Gelucire^®^, which also present peaks at between 20–25° 2θ [33]. A third peak of lower intensity can also be distinguished at around 6.8° 2θ, which is more clearly visible in G39 (Figure 5). The spectra observed in the granules is a mixture of the spectra obtained with their component raw materials, and the lower the proportion of each material in the composition of the granules, the lower the intensity of the characteristic peaks (Figure 5). 

In conclusion, this analysis allows us to confirm that no drug-Gelucire^®^ interaction occurs in the granulation process.

However, various changes were observed in TFV in DSC and HSM that have been attributed to the crystalline transition of the drug, although a further study was done to confirm this.

The X-Ray thermodiffraction technique revealed the changes in the drug’s crystallinity caused by temperature. A study was done to confirm that the changes observed in DSC and HSM at 167 °C and 220 °C correspond to the crystalline transition of TFV. This consisted of measuring a sample of pure TFV by X-ray diffraction at room temperature (25 °C) and then after heating. The temperature was maintained for five minutes before and after the temperatures at which changes were observed, then a measurement was taken by X-ray diffraction. After reaching 225 °C, higher than the temperatures at which the previous change had occurred, the sample was cooled and new measurements were taken using the same procedure to determine whether the temperature-related changes are reversible.

**Figure 6:**
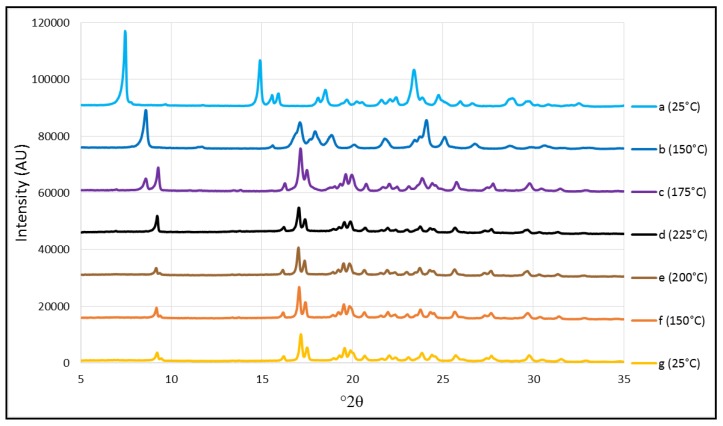
X-ray thermodiffraction patterns of pure TFV while heating to 25 °C (a), 150 °C (b), 175 °C (c) and 225 °C (d); and then while cooling to 200 °C (e), 150 °C (f) and 25 °C (g).

The spectra observed when the sample was analyzed at 25 °C is exactly the same as in the previous study at room temperature (Figure 5 and Figure 6). Although other authors have found that the crystallinity of TFV does not change with temperature, their studies reached only 80 °C [32,34]. In our study, the sample was heated to 150 °C before taking the second measurement, prior to the first change in the structure of TFV. Surprisingly, changes can be seen in TFV at this temperature. This is probably because the first transition has already begun, as the characteristic peaks have been displaced to 7.3° and 23.4° 2θ, the peak that was present at 14.7° 2θ has disappeared, and a series of peaks have appeared between 16–19° 2θ. At 175 °C, the spectrum is completely different to the one obtained at 25 °C, with a double peak between 8–9° 2θ, another two double peaks around 17–18° 2θ, and a band with multiple peaks between 19–20° 2θ that were not observed at lower temperatures. This clearly confirms that the first crystalline transition of TFV is complete (Figure 6). At 225 °C the spectrum is slightly different: The peak at 9° 2θ disappears and the intensity of the peak at 17° 2θ decreases. The second crystalline change is therefore complete at 225 °C, allowing the dissolution of TFV in its new state in the melted Gelucire^®^, as observed in the HSM studies. Finally, the sample was cooled and new measurements were taken, and no change was observed in X-ray diffraction, thus confirming that the crystalline changes in TFV are irreversible.

After the IR, thermal and X-ray analysis of the prepared granules, we can confirm that TFV is not altered in the hot-melt granulation process but conserves its therapeutic properties, thus enabling the use of the granules for prevention of HIV.

### 3.2. Assessment of the Tablets

#### 3.2.1. Swelling Behavior

The evaluation of the swelling behavior of the formulations has a twofold importance: The capture of water is the main mechanism controlling the release of the drug, since it is unable to diffuse until the hydrophilic polymer swells and the water surrounds the granules [35]; and the swelling of the formulations can be seen as a way of improving adherence to prophylactic treatment, as the lower water capture would make the tablets more comfortable for women.

**Figure 7:**
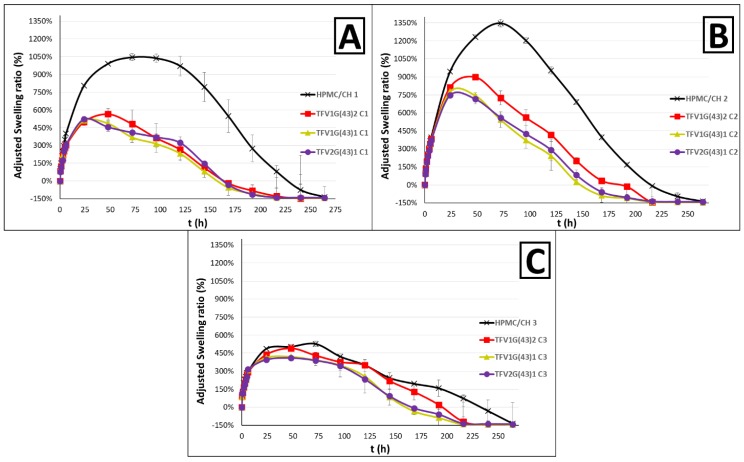
Adjusted swelling ratio profiles obtained from batches containing TFV/G43 drug-loaded granules in HPMC/CH matrices with equal ratios of both polymers (**A**); more HPMC than CH (**B**); and more CH than HPMC (**C**). They are all compared with standard formulations of HPMC/CH [16].

The inclusion of the drug granulated with G43 in the polymer matrix clearly improves the formulation in terms of swelling. As can be seen in Figure 7, the maximum swelling ratio is notably lower than in the formulations containing the drug without Gelucire^®^ [16], and especially in the formulations in which HPMC is greater than or equal to CH, due to the predominantly hydrophobic character of Gelucire^®^. The formulation achieves complete erosion sooner with the addition of the granules: After 216 h instead of the 264 h for the original formulation. This behavior would imply greater adherence to prophylaxis, since the formulation would remain less time in the vaginal environment after the drug release, making it more comfortable for women. The more HPMC there is in the formulation, the higher the maximum swelling ratio. This was expected, as the swelling capacity of HPMC is higher than CH [24]. Finally, no clear differences ca be seen between the formulations containing granules with a different G43/TFV ratio (Figure 7).

**Figure 8:**
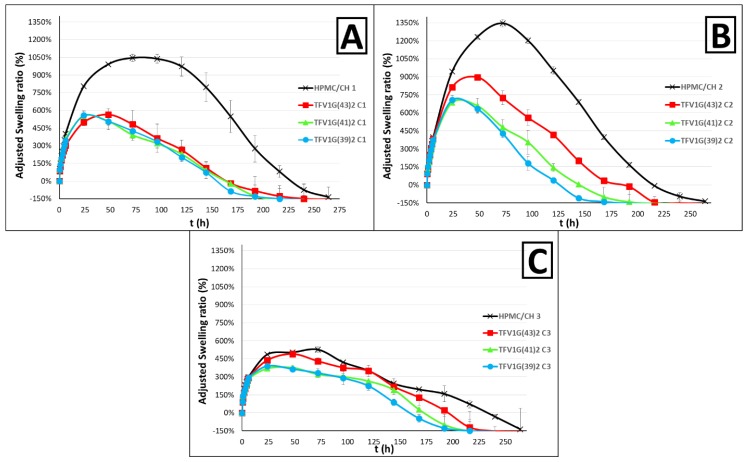
Adjusted swelling ratio profiles obtained from batches containing TFV/Gelucire^®^ (in a 1:2 ratio) granules in HPMC/CH matrices with equal ratios of both polymers (**A**); more HPMC than CH (**B**); and more CH than HPMC (**C**). They are all compared with standard formulations of HPMC/CH [16].

The use of different G39 or a mixture of G39 and G43 does not appear to change the swelling behavior of the formulations. This is to be expected since HPMC and CH are polymers with a capacity to capture water, and both Gelucire^®^ act similarly as a barrier to water diffusion. Only the tablets with more HPMC than CH show a slight difference in the formulations containing G39 (or a G39/G43 mixture), which have a lower water capture than tablets with only G43. Nevertheless, an improvement is again observed compared to the formulations without Gelucire^®^ (Figure 8).

The inclusion of the granules creates barriers within the tablet that hinder the diffusion of water and leads to lower water capture. This not only makes the tablets more comfortable, but would also probably prevent the loss of the drug, since it contains less water through which to diffuse. We can therefore formulate the hypothesis that this formulation would be more effective for the controlled release of TFV, although this must be confirmed in subsequent drug-release studies.

#### 3.2.2. Release Study

**Figure 9:**
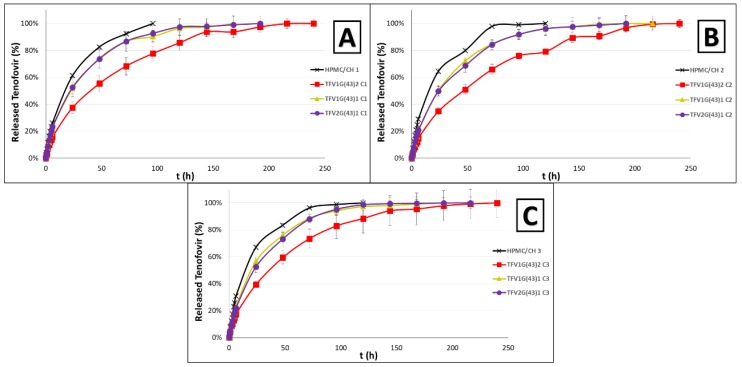
TFV release profiles obtained from batches including TFV/G43 drug-loaded granules in HPMC/CH matrices with equal ratios of both polymers (**A**), more HPMC than CH (**B**) and more CH than HPMC (**C**). All of them are compared with standard formulations of HPMC/CH [16].

As can be seen in Figure 9, the inclusion of mixed TFV/G43 granules in the formulations enhances the controlled release of the drug. These differences can be observed from the beginning of the drug release test, as the Gelucire^®^ must soften as it forms granules with the drug to allow the release of the TFV. This softening is enabled by the presence of water and body temperature. Once the Gelucire^®^ is softened and the drug diffuses through it, it is incorporated in a matrix of HPMC and CH polymers, which form a gel in the vaginal medium through which the drug must diffuse. As has been observed in the swelling test, the presence of the granules hinders the swelling of the polymers, which also causes the drug to be released from the tablet in a more sustained way. 

In the samples containing granules with equal or more TFV than G43 (TFV2G(43)1 and TFV1G(43)1), the TFV drug release profiles obtained are quite similar. In these samples the main improvement is the slowdown of the release of TFV, since almost all the drug is released at 120h (as occurred in the samples that do not use Gelucire^®^), although there are significant differences in the drug release behavior. In batches with TFV1G(43)2 granules, which include twice the amount of G43 as TFV, the improvement is much more marked (as an example, at 24 h approximately half the amount of drug has been released as in standard batches). This is because of the higher amount of G43 impedes softening and it takes longer for the drug to be released from the granules. Drug release is prolonged to 216–240 h, thus achieving 9−10 days of a controlled release of TFV. This is double the time taken without Gelucire^®^ granules, and could represent a milestone in HIV transmission, since a degradable vaginal formulation capable of reaching these controlled release times has never previously been achieved.

**Figure 10:**
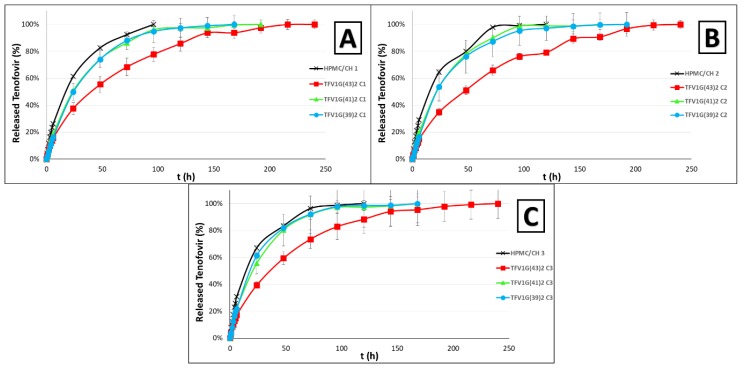
TFV release profiles obtained from batches containing TFV/Gelucire^®^ (in a ratio of 1:2) granules in HPMC/CH matrices with equal ratios of both polymers (**A**); more HPMC than CH (**B**); and more CH than HPMC (**C**). All are compared with standard formulations of HPMC/CH [16].

It was also necessary to evaluate whether a Gelucire^®^ with a melting point that was closer to body temperature would alter the release of the drug. Figure 10 shows that when G39 or a mixture of G39 and G41 is used to manufacture the granules, TFV is released faster than in tablets with TFV/G43 granules. These batches confirm that the softening of the Gelucire^®^ is a crucial factor for controlling TFV release; this agrees with other authors, who also highlight that in the case of Gelucire^®^ that melt above body temperature, the release of the drug depends on the composition and HLB value [36].

The TFV1G(43)2 granules are therefore undoubtedly the best formulation. Finally, a comparison must be made of the formulations containing different proportions of HPMC/CH. Although the three proportions have similar TFV release profiles, the formulation with the most HPMC (TFV1G(43)2 C2) shows the greatest controlled release (Figure 10B).

Although the figures visually identify the improvements achieved with the formulations, the *f*_2_ similarity factor was also calculated as a statistical analysis that could demonstrate these differences [37]. Table 3 shows the *f*_2_ values for all the formulations.

The first point to emphasize is that all the formulations developed in the present work improve those with the same proportion of HPMC/CH but without Gelucire^®^ (except for formulation TFV2G(43)1 C1, which has a slight similarity with the formulation without Gelucire^®^). This highlights the role of Gelucire^®^ in controlling the release of TFV from the tablets. However, when comparing formulations with the same granules but with a different proportion of the HPMC/CH mixture, significant differences were observed in only one case (batches TFV1G(39)2 C1 and TFV1G(39)2 C3). This reaffirms the fact that the combination of HPMC and CH forms a very robust mixed gel capable of delaying the release of TFV regardless of the proportion of polymer [16]. Although the granules’ ability to control the release has been demonstrated, these results must be compared with formulations with the same proportion of HPMC/CH but different granules, in order to determine which granules are the most suitable. The comparison of these formulations confirms that the granules identified in Figure 9 as best controlling TFV release (TFV1G(43)2) always present significant differences with the other granules, regardless of the proportion of HPMC/CH in the tablets that include the granules.

The statistical analysis therefore shows that the TFV1G(43)2 granules are the best option for preparing our formulations, although it is impossible to specify the most appropriate proportion of hydrophilic polymers to include in these granules since they are all very similar.

Finally, the experimental data obtained in the drug release tests were adjusted to different mathematical models (zero order, first order, Higuchi, Hixson-Crowell, Hopfenberg and Korsmeyer-Peppas). The correlation coefficients (*r*^2^) obtained after the adjustment are shown in Table 4.

As can be seen, all the formulations have a similar drug release profile and a good fit to the kinetics of Higuchi, Hixson-Crowell, Hopfenberg and Korsmeyer-Peppas (Table 4). However, there are slight differences between the batches that merit discussion. One observation is that all batches containing G43 tend to have a better fit to Higuchi kinetics. This model is often used to describe the behavior of matrix tablets, where the drug is released through a diffusion mechanism [38]. The most likely reason that these batches fit this kinetic more closely is that the G43 softens slowly, and the gelation of the outer layer of the hydrophilic polymer has already taken place when it occurs. This explains why the drug release is predominantly controlled by the diffusion mechanism (through both the softened Gelucire^®^ and the gel formed by the HPMC/CH mixture).

The tablets containing the mixture of G43 and G39 have a better fit to the Hixson-Crowell kinetics, a mathematical model applied when the drug is released from the parallel planes of the dosage form, which maintains its shape while its size decreases [39]. In these cases the Gelucire^®^ mixture softens a little faster than in the previous ones; one possible explanation for this drug-release mechanism is that the hydrophilic polymers gel from the outer to the inner layers, and the Gelucire^®^ softens as the vaginal fluid reaches the granules through the gel. This explains why the drug is released in layers, first diffusing the TFV in the outer layers of the tablet, and then the drug in the innermost layers.

Finally, batches with G39 are better fitted to the Korsmeyer-Peppas kinetics (except batch TFV1G(39)2 C1, which has a better fit to the Hixson-Crowell kinetics, although it also fits the Korsmeyer-Peppas kinetics quite well) (Table 4). The Korsmeyer-Peppas model gives us the most information about the drug-release mechanism, since the drug is released in different ways depending on the value of n obtained in the adjustment [40]. Thus for cylindrical tablets, TFV release follows a pure diffusion process (*n* ≤ 0.45) or an anomalous transport with simultaneous structural modification and diffusion (0.45 < *n* < 0.89). If there is a structural modification of the polymer matrix, the release is classified as transport case II (*n* = 0.89) or transport Supercase II (*n* > 0.89).

In our case, almost all the formulations can be seen to follow a simultaneous mechanism of structural modification and diffusion (Table 5), which is to be expected since it is first necessary for the hydrophilic polymer gel and the Gelucire^®^ to soften (structural modification) and the TFV to subsequently diffuse before the drug can be released. However, there are three exceptions which present a Supercase II release and are precisely the three that include G39 in the granules (Table 5). In these cases, the melting temperature of the vehicle is so low that the G39 softens as soon as it is at body temperature, even before the vaginal fluid reaches the granules, implying that the structure of the tablet changes even before the HPMC/CH mixture begins to gel.

In addition to providing an insight into the mechanism of TFV release, this adjustment allows us to identify which formulation best controls the drug release based on the values of the release constants (*K_H_*, *K_HC_*, *K_HF_* and *K_KP_*). A comparison of these constants conclusively highlights the batches with the TFV1(G43)2 granules previously identified as the most suitable for controlled-release formulations. Taking the Higuchi kinetic as an example, which has the best fit for these formulations (Table 4), the batches that include these granules have a *K_H_* value of 0.08, while the *K_H_* value of the other batches is always greater than 0.1 (Table 5). This points to the greater control over the release of the TFV from these batches, but once again makes it impossible to select the ideal proportion of HPMC/CH, given the close similarity of the constants obtained with batches C1, C2 and C3.

#### 3.2.3. Mucoadhesion Test

Finally, another crucial parameter to evaluate was the mucoadhesion residence time of the formulations. Mucoadhesion is important because the tablets must remain adhered to the vaginal mucosa during the time TFV is being released [41]. The detachment of the formulation would imply an incomplete prophylactic treatment and discomfort for women, leading to a decrease in the use of the formulations.

**Figure 11:**
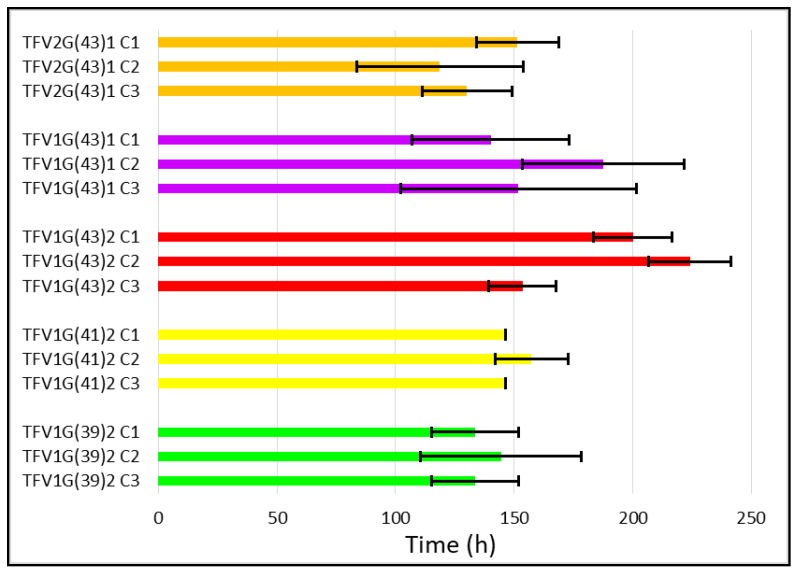
Mucoadhesion residence time in the simulated vaginal fluid in the batches.

A comparison of formulations with different ratios of TFV/G43 reveals that the more G43 the formulation contains, the longer it remains adhered to the vaginal mucosa (Figure 11). As this excipient has no adhesive properties this bioadhesion cannot be directly attributed to G43, so the only possible explanation is that it acts as a structural agent that helps form a more robust gel—as observed in the swelling test (Figure 7)—and hence remains attached for longer. In terms of the influence of the type of Gelucire^®^, it can be seen that the lower the melting point of the Gelucire^®^, the shorter the mucoadhesion time. This is probably due to the easier and faster softening of G39, which again causes the tablets to erode faster. Finally, the C2 batches appear to be the most mucoadhesive, undoubtedly due to the higher mucoadhesion of HPMC than CH [24]. 

TFV1G(43)2 granules therefore confer the strongest adhesive properties on the tablets. Among these batches, it is worth highlighting TFV1G(43)2 C2, which remains adhered to the vaginal mucosa for almost 225 h.

**Figure 12:**
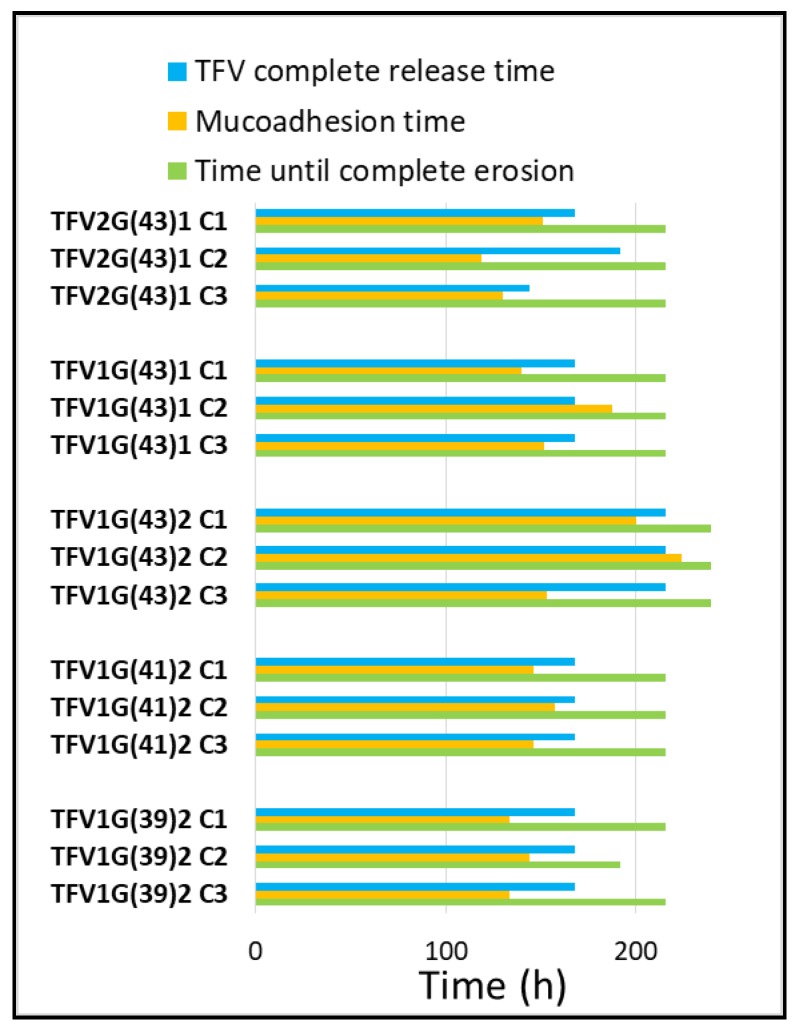
Summary of the data obtained from the drug release, mucoadhesion and swelling test. TFV complete release time (blue), mucoadhesion time (orange) and time until complete erosion (green) are shown for each batch.

The results of these studies, which are summarized in Figure 12, can be used to select the optimal formulation for preventing the sexual transmission of HIV.

The main parameter is undoubtedly the time taken to release the TFV from the formulation, since the aim of this work was to develop weekly sustained-release tablets whose administration could be markedly spaced in order to improve women’s adherence to prophylactic treatment, which is currently the main problem with vaginal microbicides. Batches containing TFV1G(43)2 granules achieve the longest controlled release of TFV (216h), above the established target. However, the objective was not only to achieve a longer release time, but also to ensure the mucoadhesion of the formulation for as long as the drug is being released; there are only two formulations whose mucoadhesion time exceeds their complete TFV release time (batches TFV1G(43)1 C2 and TFV1G(43)2 C2). Finally, the formulation must not remain for long in the vaginal environment after the complete TFV release. 

Batch TFV1G(43)2 C2 is conclusively selected as the optimal formulation for HIV prevention, since it is able to release TFV in a sustained manner for 216 h and remains attached to the vaginal mucosa throughout. Almost all the formulation has been eroded by the end of the process (with complete disintegration at 240 h), making it comfortable for women and enabling the next dose of the prophylactic treatment to be easily administered.

## 4. Conclusions

The combination of hydrophobic granules (prepared with Gelucire^®^) and hydrophilic matrices (HPMC and CH) in the development of vaginal mucoadhesive tablets allow the sustained-release of TFV.

The ratio TFV-Gelucire, as well as the proportion of HPMC and CH is critical for the development of the optimal formulation. Thus, the one with the best results contains TFV1G(43)2 granules in a mixture of HPMC and CH in a ratio of 1.9:1, which allows the sustained release of TFV for 216h. The formulation remains adhered to the vaginal mucosa throughout this time, so a weekly administration of the tablets could protect women again the sexual transmission of HIV.

## Figures and Tables

**Table 1:** Granule composition (mg).

Granules	Tenofovir	Gelucire^®^ 39	Gelucire^®^ 43
TFV-1-G43-2	30		60
TFV-1-G43-1	30		30
TFV-2-G43-1	30		15
TFV-1-G41-2	30	30	30
TFV-1-G39-2	30	60	

**Table 2:** Tablet composition (mg/unit).

Batch	HPMC	CH	ACDP	PVP	TFV	G43	G39	MgSt
TFV-1-G43-2 C1	145	145	45	27	30	60		3
TFV-1-G43-2 C2	190	100	45	27	30	60		3
TFV-1-G43-2 C3	100	190	45	27	30	60		3
TFV-1-G43-1 C1	145	145	45	27	30	30		3
TFV-1-G43-1 C2	190	100	45	27	30	30		3
TFV-1-G43-1 C3	100	190	45	27	30	30		3
TFV-2-G43-1 C1	145	145	45	27	30	15		3
TFV-2-G43-1 C2	190	100	45	27	30	15		3
TFV-2-G43-1 C3	100	190	45	27	30	15		3
TFV-1-G41-2 C1	145	145	45	27	30	30	30	3
TFV-1-G41-2 C2	190	100	45	27	30	30	30	3
TFV-1-G41-2 C3	100	190	45	27	30	30	30	3
TFV-1-G39-2 C1	145	145	45	27	30		60	3
TFV-1-G39-2 C2	190	100	45	27	30		60	3
TFV-1-G39-2 C3	100	190	45	27	30		60	3

HPMC: Hydroxypropylmethylcellulose; CH: Chitosan; ACDP: Anhydrous calcium hydrogen phosphate.

**Table 3:** Similarity factor (*f*_2_) values for the release profiles obtained from reference and problem formulations. Comparisons with significant difference (*f*_2_ < 65) are in bold.

REFERENCE	PROBLEM	*f*_2_	REFERENCE	PROBLEM	*f*_2_	REFERENCE	PROBLEM	*f*_2_
C1	T1G(43)2 C1	**44.6**	C1	C2	84.3	T1G(43)2 C1	T1G(43)2 C2	83.4
C1	T1G(43)1 C1	**64.7**	C1	C3	76.9	T1G(43)2 C1	T1G(43)2 C3	78.4
C1	T2G(43)1 C1	66.9	C2	C3	85.6	T1G(43)2 C2	T1G(43)2 C3	68.1
C1	T1G(41)2 C1	**60.4**	T1G(43)2 C1	T1G(43)1 C1	**54.5**	T1G(43)1 C1	T1G(43)1 C2	83.5
C1	T1G(39)2 C1	**60.5**	T1G(43)2 C1	T2G(43)1 C1	**53.7**	T1G(43)1 C1	T1G(43)1 C3	84.9
C2	T1G(43)2 C2	**40.9**	T1G(43)2 C1	T1G(41)2 C1	**56.3**	T1G(43)1 C2	T1G(43)1 C3	75.1
C2	T1G(43)1 C2	**55.8**	T1G(43)2 C1	T1G(39)2 C1	**57.2**	T2G(43)1 C1	T2G(43)1 C2	81.3
C2	T2G(43)1 C2	**56.7**	T1G(43)2 C2	T1G(43)1 C2	**50.0**	T2G(43)1 C1	T2G(43)1 C3	94.5
C2	T1G(41)2 C2	**60.5**	T1G(43)2 C2	T2G(43)1 C2	**51.0**	T2G(43)1 C2	T2G(43)1 C3	84.8
C2	T1G(39)2 C2	**54.7**	T1G(43)2 C2	T1G(41)2 C2	**48.4**	T1G(41)2 C1	T1G(41)2 C2	86.2
C3	T1G(43)2 C3	**43.3**	T1G(43)2 C2	T1G(39)2 C2	**50.0**	T1G(41)2 C1	T1G(41)2 C3	73.7
C3	T1G(43)1 C3	**60.5**	T1G(43)2 C3	T1G(43)1 C3	**54.6**	T1G(41)2 C2	T1G(41)2 C3	83.7
C3	T2G(43)1 C3	**56.5**	T1G(43)2 C3	T2G(43)1 C3	**59.1**	T1G(39)2 C1	T1G(39)2 C2	88.1
C3	T1G(41)2 C3	**61.9**	T1G(43)2 C3	T1G(41)2 C3	**52.4**	T1G(39)2 C1	T1G(39)2 C3	**62.7**
C3	T1G(39)2 C3	**64.1**	T1G(43)2 C3	T1G(39)2 C3	**49.3**	T1G(39)2 C2	T1G(39)2 C3	67.4

**Table 4:** Correlation coefficients obtained when experimental data are fitted to different mathematical models.

Batch	Correlation Coefficients (*r*^2^)					
	Zero Order	First Order	Higuchi	Hixson-Crowell	Hopfenberg	Korsmeyer-Peppas
TFV1G(43)2 C1	0.9557	0.7162	0.9975	0.9888	0.9824	0.9915
TFV1G(43)2 C2	0.9658	0.7139	0.9961	0.9925	0.9877	0.9876
TFV1G(43)2 C3	0.9567	0.7188	0.9971	0.9922	0.9858	0.9911
TFV1G(43)1 C1	0.9140	0.6436	0.9885	0.9749	0.9624	0.9795
TFV1G(43)1 C2	0.9298	0.6610	0.9911	0.9884	0.9771	0.9887
TFV1G(43)1 C3	0.9110	0.6514	0.9878	0.9835	0.9690	0.9896
TFV2G(43)1 C1	0.9190	0.6474	0.9920	0.9852	0.9723	0.9801
TFV2G(43)1 C2	0.9351	0.6671	0.9954	0.9911	0.9813	0.9864
TFV2G(43)1 C3	0.9306	0.6849	0.9939	0.9938	0.9834	0.9933
TFV1G(41)2 C1	0.9377	0.663	0.9930	0.9958	0.9877	0.9850
TFV1G(41)2 C2	0.9307	0.6604	0.9912	0.9963	0.9897	0.9889
TFV1G(41)2 C3	0.9148	0.6139	0.9886	0.9918	0.9775	0.9662
TFV1G(39)2 C1	0.9384	0.6522	0.9886	0.9948	0.9850	0.9862
TFV1G(39)2 C2	0.9270	0.6708	0.9854	0.9916	0.9796	0.9941
TFV1G(39)2 C3	0.9005	0.6437	0.9819	0.9879	0.9705	0.9983

**Table 5:** TFV release kinetics from the different batches showing the kinetic constants for the models with the best fit.

Batch	Higuchi	Hixson-Crowell	Hopfenberg	Korsmeyer-Peppas	
	*K_H_*	*K_HC_*	*K_HF_*	*K_KP_*	*n*
TFV1G(43)2 C1	0.083	0.0040	0.0054	0.042	0.68
TFV1G(43)2 C2	0.080	0.0039	0.0052	0.036	0.71
TFV1G(43)2 C3	0.088	0.0045	0.0060	0.046	0.67
TFV1G(43)1 C1	0.102	0.0059	0.0075	0.045	0.82
TFV1G(43)1 C2	0.103	0.0060	0.0076	0.037	0.86
TFV1G(43)1 C3	0.106	0.0065	0.0081	0.048	0.81
TFV2G(43)1 C1	0.103	0.0061	0.0077	0.056	0.73
TFV2G(43)1 C2	0.108	0.0058	0.0074	0.047	0.78
TFV2G(43)1 C3	0.105	0.0065	0.0081	0.052	0.75
TFV1G(41)2 C1	0.107	0.0067	0.0083	0.038	0.85
TFV1G(41)2 C2	0.111	0.0076	0.0091	0.040	0.86
TFV1G(41)2 C3	0.110	0.0073	0.0089	0.043	0.88
TFV1G(39)2 C1	0.108	0.0066	0.0083	0.026	0.97
TFV1G(39)2 C2	0.109	0.0067	0.0084	0.029	0.93
TFV1G(39)2 C3	0.112	0.0075	0.0091	0.042	0.93

## References

[1] Chaudhary R.S., Amankwaa E., Kumar S., Hu T., Chan M., Sanghvi P. (2015). Application of a hot-melt granulation process to enhance fenofibrate solid dose manufacturing. Drug Develop. Ind. Pharm..

[2] Jakson K.A., Hunt J.D. (1966). Binary eutectic solidification. Trans Met Soc AIME.

[3] Patil H., Tiwari R.V., Repka M.A. (2015). Hot-Melt Extrusion: From Theory to Application in Pharmaceutical Formulation. AAPS PharmSciTech.

[4] Chen Y.-C., Ho H.-O., Chiou J.-D., Sheu M.-T. (2014). Physical and dissolution characterization of cilostazol solid dispersions prepared by hot melt granulation (HMG) and thermal adhesion granulation (TAG) methods. Int. J. Pharm..

[5] Verhoeven E., De Beer T., Mooter G.V.D., Remon J., Vervaet C. (2008). Influence of formulation and process parameters on the release characteristics of ethylcellulose sustained-release mini-matrices produced by hot-melt extrusion. Eur. J. Pharm. Biopharm..

[6] Özgüney I., Shuwisitkul D., Bodmeier R. (2009). Development and characterization of extended release Kollidon^®^ SR mini-matrices prepared by hot-melt extrusion. Eur. J. Pharm. Biopharm..

[7] Verstraete G., Mertens P., Grymonpré W., Van Bockstal P.-J., De Beer T., Boone M.N., Van Hoorebeke L., Remon J., Vervaet C. (2016). A comparative study between melt granulation/compression and hot melt extrusion/injection molding for the manufacturing of oral sustained release thermoplastic polyurethane matrices. Int. J. Pharm..

[8] Almeida A., Saerens L., De Beer T., Remon J., Vervaet C. (2012). Upscaling and in-line process monitoring via spectroscopic techniques of ethylene vinyl acetate hot-melt extruded formulations. Int. J. Pharm..

[9] El Hadri M., Achahbar A., El Khamkhami J., Khelifa B., Faivre V., Abbas O., Bresson S. (2016). Lyotropic behavior of Gelucire 50/13 by XRD, Raman and IR spectroscopies according to hydration. Chem. Phys. Lipids.

[10] Chambin O., Jannin V. (2005). Interest of Multifunctional Lipid Excipients: Case of Gelucire^®^ 44/14. Drug Develop. Ind. Pharm..

[11] Upadhyay P., Pandit J.K., Wahi A.K. (2013). Gelucire: An alternative formulation technological tool for both sustained and fast release of drugs in treating diabetes mellitus type II disease. J. Sci. Ind. Res..

[12] UNAIDS When Women Lead Change Happens. http://www.unaids.org/sites/default/files/media_asset/when-women-lead-change-happens_en.pdf.

[13] Aurpibul L., Puthanakit T. (2015). Review of Tenofovir Use in HIV-infected Children. Pediatric Infect. Dis. J..

[14] Mertenskoetter T., Kaptur P. (2011). Update on microbicide research and development-seeking new HIV prevention tools for women. Eur. J. Med Res..

[15] Hankins C.A., Dybul M.R. (2013). The promise of pre-exposure prophylaxis with antiretroviral drugs to prevent HIV transmission: A review. Curr. Opin. HIV AIDS.

[16] Notario-Pérez F., Cazorla-Luna R., Martín-Illana A., Ruiz-Caro R., Tamayo A., Rubio J., Veiga M.-D. (2018). Optimization of tenofovir release from mucoadhesive vaginal tablets by polymer combination to prevent sexual transmission of HIV. Carbohydr. Polym..

[17] Cazorla-Luna R., Notario-Pérez F., Martín-Illana A., Ruiz-Caro R., Tamayo A., Rubio J., Veiga M.D. (2019). Chitosan-Based Mucoadhesive Vaginal Tablets for Controlled Release of the Anti-HIV Drug Tenofovir. Pharmaceutics.

[18] Ruiz-Caro R., Veiga-Ochoa M.D. (2009). Characterization and Dissolution Study of Chitosan Freeze-Dried Systems for Drug Controlled Release. Molecules.

[19] Owen D.H., Katz D.F. (1999). A vaginal fluid simulant. Contraception.

[20] Sánchez-Sánchez M.-P., Martín-Illana A., Ruiz-Caro R., Bermejo P., Abad M.-J., Carro R., Bedoya L.-M., Tamayo A., Rubio J., Fernández-Ferreiro A. (2015). Chitosan and Kappa-Carrageenan Vaginal Acyclovir Formulations for Prevention of Genital Herpes. In Vitro and Ex Vivo Evaluation. Mar. Drugs.

[21] Moore J.W., Flanner H.H. (1996). Mathematical comparison of dissolution profiles. Pharma Tec..

[22] Mamani P.L., Ruiz-Caro R., Veiga M.D. (2012). Matrix Tablets: The Effect of Hydroxypropyl Methylcellulose/Anhydrous Dibasic Calcium Phosphate Ratio on the Release Rate of a Water-Soluble Drug Through the Gastrointestinal Tract I. In Vitro Tests. AAPS PharmSciTech.

[23] Dash S., Murthy P.N., Nath L., Chowdhury P. (2010). Kinetic modeling on drug release from controlled drug delivery systems. Acta Pol. Pharm..

[24] Notario-Pérez F., Martín-Illana A., Cazorla-Luna R., Ruiz-Caro R., Bedoya L.-M., Tamayo A., Rubio J., Veiga M.-D., Harding D., Sashiwa H. (2017). Influence of Chitosan Swelling Behaviour on Controlled Release of Tenofovir from Mucoadhesive Vaginal Systems for Prevention of Sexual Transmission of HIV. Mar. Drugs.

[25] Ochiuz L., Grigoras C., Popa M., Stoleriu I., Munteanu C., Timofte D., Profire L., Grigoras A.G. (2016). Alendronate-Loaded Modified Drug Delivery Lipid Particles Intended for Improved Oral and Topical Administration. Molecules.

[26] Eloy J.D.O., Saraiva J., De Albuquerque S., Marchetti J.M., Albuquerque S. (2012). Solid Dispersion of Ursolic Acid in Gelucire 50/13: A Strategy to Enhance Drug Release and Trypanocidal Activity. AAPS PharmSciTech.

[27] Patil S., Kadam C., Pokharkar V. (2017). QbD based approach for optimization of Tenofovir disoproxil fumarate loaded liquid crystal precursor with improved permeability. J. Adv. Res..

[28] Ramkumaar G.R., Srinivasan S., Bhoopathy T.J., Gunasekaran S. (2013). Vibrational Spectroscopic Studies of Tenofovir Using Density Functional Theory Method. J. Chem..

[29] Svensson A., Neves C., Cabane B. (2004). Hydration of an amphiphilic excipient, Gelucire^®^ 44/14. Int. J. Pharm..

[30] National Center for Biotechnology Information PubChem Compound Database, CID=464205. https://pubchem.ncbi.nlm.nih.gov/compound/464205.

[31] Veiga M., Bernad M., Escobar C. (1993). Thermal behaviour of drugs from binary and ternary systems. Int. J. Pharm..

[32] Agrahari V., Putty S., Mathes C., Murowchick J.B., Youan B.-B.C. (2014). Evaluation of degradation kinetics and physicochemical stability of tenofovir. Drug Test. Analysis.

[33] Albarahmieh E., Qi S., Craig D.Q. (2016). Hot melt extruded transdermal films based on amorphous solid dispersions in Eudragit RS PO: The inclusion of hydrophilic additives to develop moisture-activated release systems. Int. J. Pharm..

[34] Zhang T., Zhang C., Agrahari V., Murowchick J.B., Oyler N.A., Youan B.B.C. (2013). Spray drying tenofovir loaded mucoadhesive and pH-sensitive microspheres intended for HIV prevention. Antivir. Res..

[35] Yin X., Li H., Guo Z., Wu L., Chen F., De Matas M., Shao Q., Xiao T., York P., He Y. (2013). Quantification of Swelling and Erosion in the Controlled Release of a Poorly Water-Soluble Drug Using Synchrotron X-ray Computed Microtomography. AAPS J.

[36] Geeta M.P., Madhabhai M.P. (2009). Design and in vitro evaluation of a novel vaginal drug delivery system based on gelucire. Curr Drug Deliv.

[37] Stevens R.E., Gray V., Dorantes A., Gold L., Pham L. (2015). Scientific and Regulatory Standards for Assessing Product Performance Using the Similarity Factor, f2. AAPS J.

[38] Paul D. (2011). Elaborations on the Higuchi model for drug delivery. Int. J. Pharm..

[39] Hixson A.W., Crowell J.H. (1931). Dependence of reaction velocity upon surface and agitation: Theoretical considerations. Ind Eng Chem..

[40] Korsmeyer R.W., Gurny R., Doelker E., Buri P., Peppas N.A. (1983). Mechanisms of solute release from porous hydrophilic polymers. Int. J. Pharm..

[41] Caramella C.M., Rossi S., Ferrari F., Bonferoni M.C., Sandri G. (2015). Mucoadhesive and thermogelling systems for vaginal drug delivery. Adv. Drug Deliv. Rev..

